# Algorithmic Management for Improving Collective Productivity in Crowdsourcing

**DOI:** 10.1038/s41598-017-12757-x

**Published:** 2017-10-02

**Authors:** Han Yu, Chunyan Miao, Yiqiang Chen, Simon Fauvel, Xiaoming Li, Victor R. Lesser

**Affiliations:** 1 Joint NTU-UBC Research Centre of Excellence in Active Living for the Elderly (LILY), Nanyang Technological University, Singapore, 639798 Singapore; 2 School of Computer Science and Engineering, Nanyang Technological University, Singapore, 639798 Singapore; 3 School of Computer and Control Engineering, University of Chinese Academy of Sciences, Beijing, 100049 China; 4 Institute of Computing Technology, Chinese Academy of Sciences, Beijing, 100190 China; 5 Institute of Network Computing and Information Systems, Peking University, Beijing, 100871 China; 6 School of Computer Science, University of Massachusetts Amherst, Amherst, Massachusetts 01002 USA

## Abstract

Crowdsourcing systems are complex not only because of the huge number of potential strategies for assigning workers to tasks, but also due to the dynamic characteristics associated with workers. Maximizing social welfare in such situations is known to be NP-hard. To address these fundamental challenges, we propose the surprise-minimization-value-maximization (SMVM) approach. By analysing typical crowdsourcing system dynamics, we established a simple and novel *worker desirability index* (WDI) jointly considering the effect of each worker’s reputation, workload and motivation to work on collective productivity. Through evaluating workers’ WDI values, SMVM influences individual workers in real time about courses of action which can benefit the workers and lead to high collective productivity. Solutions can be produced in polynomial time and are proven to be asymptotically bounded by a theoretical optimal solution. High resolution simulations based on a real-world dataset demonstrate that SMVM significantly outperforms state-of-the-art approaches. A large-scale 3-year empirical study involving 1,144 participants in over 9,000 sessions shows that SMVM outperforms human task delegation decisions over 80% of the time under common workload conditions. The approach and results can help engineer highly scalable data-driven algorithmic management decision support systems for crowdsourcing.

## Introduction

Crowdsourcing systems refer to systems designed to obtain needed services, ideas, or content by soliciting contributions from a large group of people and especially from the online community^1^. Over the years, crowdsourcing technologies have spawned the “sharing economy”, an economic paradigm in which individuals can borrow or rent assets owned by someone else. Examples include Uber (https://www.uber.com), the world’s largest “taxi company” which crowdsources rides for commuters, and Airbnb (https://www.airbnb.com), the world’s largest “hotel chain” which crowdsources accommodations for travellers. A recent study by PricewaterhouseCoopers (PwC) estimates that the size of the sharing economy will reach $335 billion by 2025^2^. Nowadays, many organisations flexibly outsource work to a global pool of workers on a temporary basis. Indeed, crowdsourcing has been used successfully for a range of tasks, including collaborative sensing^3–9^, citizen science^10,11^, and human-powered online security^12^.

In crowdsourcing systems, crowdsourcers propose tasks for workers to complete. Such systems often consist of a large number of interacting crowdsourcers and workers. There are typically multiple workers (i.e. resources) available for a crowdsourcer. For example, in Amazon’s Mechanical Turk (mTurk) (https://www.mturk.com), workers outnumber crowdsourcers almost 20 to 1^13^. As such, the number of potential ways for the division of work among workers is very large. The cost of delegating a task to a worker in crowdsourcing does not solely depend on the number of crowdsourcers engaging this worker. As a human being, a worker is not only limited in productivity (i.e. how much workload he can process per unit time), but also has different competence which can affect the quality of the results produced^14^. These personal characteristics may vary over time and can only be estimated based on observed track records. Crowdsourcers not only aim to obtain results for tasks in a timely manner, but also care about the quality of the results. Thus, the cost functions depend on both the number of crowdsourcers engaging a worker as well as the workers’ competence.

The business objective of a crowdsourcing system is to maximize its revenue by ensuring as many tasks as possible can be completed before their deadlines with good quality. In order to achieve this goal, the collective productivity of all workers needs to be maximally utilized in an opportunistic manner in response to stochastic changes in situational factors (e.g., workload distribution, task progress, workers’ motivation to work). Optimization objective functions for modelling similar social economic systems (e.g., *Pareto* optimality^15^) are not suitable for this purpose as they normally include the concept of minimizing individual regret which interferes with the revenue maximization objective. Thus, in this paper, we adopt the social welfare maximization to collectively optimise system performance.

At the typical scale of commercial crowdsourcing systems, it has been shown that optimizing social welfare is a Non-deterministic Polynomial-time hard (NP-hard) problem^16^. The same time complexity has also been proven in alternative formulations treating crowdsourcing task delegation as a joint optimization problem, which has resulted in the need for part of the solutions to be computed offline^17^. This makes the solutions unable to keep up with changes in situational factor in real time in crowdsourcing systems. In an effort to induce high collective productivity, some crowdsourcing systems (e.g., mTurk) are incorporating algorithmic management in the form of high-level policies (e.g., reputation-based sanctioning mechanisms to induce desirable worker behaviours) into their software systems^18^. However, these approaches focus on adjusting population-level policies to signal crowdsourcers and workers in an attempt to steer the entire system. They still largely depend on how crowdsourcing participants self-organize in the face of changes in such high-level governing policies and are, thus, unable to effectively advise individual participants about the courses of action which can lead to desirable collective behaviours.

The algorithmic management research focuses on improving the quality of crowdsourcing work through accurate analytics of worker capabilities and task-worker matching^19–25^. The aim is to allow crowdsourcing systems to effectively and efficiently oversee hundreds of thousands of workers on a global scale with little human intervention. In this paper, we propose the surprise-minimization-value-maximization (SMVM) approach to address the fundamental research problem of achieving high collective productivity in crowdsourcing systems. We treat crowdsourcing task delegation as a constrained multi-objective optimization problem, and propose a formulation grounded in queueing system stability analysis^26^ in combination with recent findings in social science on apparently irrational human choice behaviours^27^ in order to coordinate task delegation decisions at scale. By analysing typical crowdsourcing system dynamics, we establish a simple and novel *worker desirability index* (WDI) (equation (10)) which uses information on workers’ reputation^28^, workload and motivation to work^29,30^ to translate system-level objectives into actionable task delegation decisions for individual workers. Instead of only maximizing the overall utility (as is the case with existing approaches^31,32^), SMVM maximizes the overall utility while minimizing the fluctuations in workers’ workloads based on the workers’ real-time WDI values.

Unlike existing approaches such as Yu *et al*.^25^, SMVM does not require additional infrastructure support apart from what currently exists in crowdsourcing systems in order to operate. It can operate in crowdsourcing systems to allocate tasks to workers with a time complexity of *O*(*N* log (*N*)), where *N* is the total number of workers in a given crowdsourcing system. In the distributed implementation of SMVM, the time complexity can be as low as *O*(1). Through analysis, we show that the performance of SMVM asymptotically approaches that of a theoretical optimal task allocation strategy with perfect foresight. SMVM has been compared to four existing task allocation approaches through extensive high resolution simulations based on a real-world dataset. SMVM is shown to be able to achieve higher quality results in shorter time compared to these approaches.

One of the problems plaguing existing algorithmic management approaches is the difficulty evaluating them with humans in the loop. The ability of the current crowdsourcing research community to propose computational approaches to improve collective productivity outstrips the ability to test them^33^. To overcome this problem, we have deployed SMVM in a serious game platform which creates various worker behaviour profiles and workload conditions so that the approach can be compared against human decision-making strategies. Between 2014 and 2016, a total of 1,144 people from diverse backgrounds have competed against SMVM in the game platform through more than 9,000 game sessions. Through this evaluation platform, SMVM is shown to outperform human strategies over 80% of the time under common workload conditions.

## Method

In this section, we formulate the aforementioned fundamental research problem of improving collective productivity in crowdsourcing systems through task delegation into a constrained multi-objective optimization problem, and propose efficient solutions to this problem.

### Deriving the Worker Desirability Index

The queueing dynamics of a worker *w*’s pending task queue can be expressed as:

1 $${q}_{w}(t+\mathrm{1)}=\,{\rm{\max }}\,[0,{q}_{w}(t)+{a}_{w}(t)-{c}_{w}(t)]$$

where *q*_*w*_(*t*) is the number of tasks in *w*’s pending task queue at the beginning of time slot *t*, *a*_*w*_(*t*) is the number of new tasks entering *w*’s pending task queue during time slot *t*, and *c*_*w*_(*t*) is the number of tasks completed by *w* during time slot *t*.

The reliability of a worker *w* at time slot *t* is a latent factor which may not be known definitively. The current prevailing practice is to estimate this value by computing *w*’s reputation, ${r}_{w}(t)\in [0,\,1]$, based on his track record. In this paper, we adopt the Extended Beta Reputation System (*BRSEXT*) method^34^. Under this method, *r*_*w*_(*t*) can be interpreted as the probability of *w* completing a task successfully. SMVM does not need to rely exclusively on the BRSEXT method. As long as the computed value for *r*_*w*_(*t*) is in the $\mathrm{[0},\,1]$ range, any reputation estimation model (e.g., ACT^35^, QASCA^24^ and others^36,37^) can be used to compute worker reputation.

Let *U*(*t*) be the expected overall utility (i.e., the sum of the expected task success rate) of a strategy which distributes tasks among a given set of *N* workers in a given way during time slot *t*. We have:

2 $$U(t)=\sum _{w=1}^{N}\,{r}_{w}(t){a}_{w}(t).$$

Recent findings in social science suggest that human choice behaviour can be accounted for by a mixture of *surprise minimization* and *value maximization* ^27^. We adopt this principle in SMVM. The variation in workload for a worker can be regarded as a form of surprise, which is to be minimized.

In SMVM, we adopt the *Lyapunov function* ^26^ to model the concept of surprise for workers. It measures the overall congestion of demand on workers at time slot *t*:

3 $$L(t)=\frac{1}{2}\,\sum _{w=1}^{N}\,{q}_{w}^{2}(t)\geqslant 0.$$

If task requests concentrate on a small number of workers, *L*(*t*) increases significantly. In essence, *L*(*t*) is the *l* ^2^-norm. The coefficient of $\tfrac{1}{2}$ is added and the $\sqrt{\cdot }$ operator is omitted to simplify the subsequent derivations without affecting the underlying meaning of the formulation.

Let **q**(*t*) be a vector of all workers’ pending task queues during time slot *t*. Using the *Lyapunov drift*, Δ(**q**(*t*)), the variation in workers’ workload can be expressed as:

4 $$\begin{array}{rcl}{\rm{\Delta }}({\bf{q}}(t)) & = & {\mathbb{E}}\,\{L(t+\mathrm{1)}-L(t)|{\bf{q}}(t)\}\\  & = & \sum _{w=1}^{N}\,(\frac{1}{2}{q}_{w}^{2}(t+\mathrm{1)}-\frac{1}{2}{q}_{w}^{2}(t))\\  & \leqslant  & \sum _{w=1}^{N}\,({q}_{w}(t){a}_{w}(t)-{q}_{w}(t){c}_{w}(t)+\frac{1}{2}[{a}_{w}^{2}(t)+{c}_{w}^{2}(t)]).\end{array}$$

In a practical crowdsourcing system, there are physical upper limits on *a*_*w*_(*t*) and *c*_*w*_(*t*) for all *w* and *t*. We denote these upper limits by ${a}_{c}^{{\rm{\max }}}$ and ${c}_{w}^{{\rm{\max }}}$. By using *θ* to denote $\tfrac{1}{2}[({a}_{w}^{{\rm{\max }}}{)}^{2}+{({c}_{w}^{{\rm{\max }}})}^{2}]$ which is non-negative, equation (4) can be re-expressed as:

5 $${\rm{\Delta }}({\bf{q}}(t))\leqslant \sum _{w=1}^{N}\,[{q}_{w}(t){a}_{w}(t)+\theta ].$$

Based on equations (2) and (5), we formulate the fundamental research problem of efficiently allocating a large number of tasks among workers as a (*utility* – *surprise*) objective function:

6 $$\frac{1}{T}\,\sum _{t=0}^{T-1}(\sigma {\mathbb{E}}\{U(t)|{\bf{q}}(t)\}-{\rm{\Delta }}({\bf{q}}(t)))\geqslant \frac{1}{T}\,\sum _{t=0}^{T-1}\sum _{w=1}^{N}({\sigma }_{w}(t)\,{r}_{w}(t)\,{a}_{w}(t)-{q}_{w}(t)\,{a}_{w}(t)-\theta )$$

where *σ*_*w*_(*t*) can be interpreted as worker *w*’s motivation to work during time slot *t*. $\sigma =\frac{1}{N}\,{\sum }_{w=1}^{w=N}\,{\sigma }_{w}(t)$ during time slot *t* is a weight factor determining the relative importance given to maximizing utility versus minimizing surprise at the system level. As the proposed SMVM approach aims to influence the new workload allocated to each worker during each time slot, ${a}_{w}(t)\in \{0,\,{{\mathbb{Z}}}^{+}\}$ is the solution variable. By considering only the terms containing the solution variables *a*_*w*_(*t*) for all workers, the objective function can be simplified as:

Maximize:

7 $$\frac{1}{T}\,\sum _{t=0}^{T-1}\,\sum _{w=1}^{N}\,{a}_{w}(t)\,[{\sigma }_{w}(t){r}_{w}(t)-{q}_{w}(t)]$$

Subject to:

8 $${r}_{w}(t)\geqslant {r}_{{\rm{\min }}},\,\forall w,\,\forall t$$

9 $${a}_{w}(t)\leqslant n{c}_{w}^{{\rm{\max }}},\,\forall w,\,\forall t$$

where *r*_min_ is the minimum reputation value a worker *w* must possess in order to be allowed to participate, and *n* > 0 is a coefficient variable which can be used to control how much new workload is allowed to enter a worker’s pending task queue at any point in time. The *r*_min_ value can be set by the crowdsourcing system administrators. The *n* value can be either set by the crowdsourcing system administrators or by each individual worker to indicate his preference.

To solve equations (7)–(9), we first propose the worker desirability index (WDI), Ψ_*w*_(*t*), which contains the terms in equation (7) other than the solution variable *a*_*w*_(*t*). The WDI is expressed as:

10 $${{\rm{\Psi }}}_{w}(t)={\sigma }_{w}(t)\,{r}_{w}(t)-{q}_{w}(t).$$

With this index, we propose an algorithm which ranks workers according to their WDIs to achieve high collective productivity in crowdsourcing systems.

### The SMVM Approach

Suppose at time slot *t*, there are a total of *Q*(*t*) new task requests which need to be allocated among *N* workers. Under the centralized implementation of SMVM, to maximize equation (7), at each time slot *t*, Algorithm 1 computes Ψ_*w*_(*t*) for every worker *w*. It then sorts all workers in descending order of their Ψ_*w*_(*t*) values. For each worker *w* who satisfies Ψ_*w*_(*t*) > 0 and Constraint (8), compute *a*_*w*_(*t*) based on Constraint (9). In this case, we adopt the average task deadline to set *n* as it can be tracked relatively easily in practice. SMVM allocates *a*_*w*_(*t*) tasks from the pool of *Q*(*t*) new tasks to worker *w* and then continues the iteration. In essence, SMVM implements the intuition that if a worker is highly reliable and currently having low workload, he or she should be assigned more tasks. The task allocation process terminates when there are no more workers with Ψ_*w*_(*t*) > 0 or when all *Q*(*t*) new task requests have been allocated. If there are any tasks not allocated, they wait to be allocated in some future time slots.

SMVM compartmentalizes considerations for different reputation under different skills by a single worker. When one type of tasks needs to be allocated, all workers are ranked with regard to their reputation in that particular domain (together with other situational factors). This process can then be repeated with regard to other types of tasks until termination conditions are reached.

**Algorithm 1:**
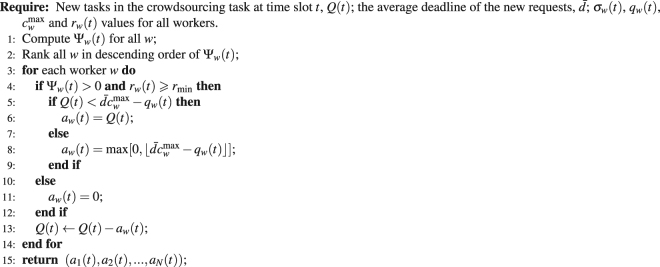
.

The time complexity of SMVM depends on the algorithm employed to rank all workers in descending order of their Ψ_*w*_(*t*) values (Line 2, Algorithm 1). Suppose mergesort^38^, with a time complexity of *O*(*N* log (*N*)), is used for this purpose. Since the time complexity of the remaining part of Algorithm 1 is *O*(*N*), the overall time complexity of Algorithm 1 is *O*(*N* log (*N*)) + *O*(*N*) = *O*(*N* log (*N*)).

In a distributed crowdsourcing system (e.g., crowdsourcing through social networks^39^), SMVM can operate through Algorithm 2. It does not allocate tasks to workers as in the case of Algorithm 1. Instead, it can advise an individual worker *w* on how many new task requests directed at him during time slot *t*, *Q*_*w*_(*t*), to accept into his pending task queue. Any task requests not accepted by *w* are returned to the crowdsourcers so that they can seek out other alternatives. Constraint (8) is not enforced by Algorithm 2, but by the task delegation approach adopted by task requesters instead (e.g., the approval rate in mTurk). As Algorithm 2 is fully distributed, its time complexity is *O*(1). Under SMVM, if a worker does not wish to participate in a crowdsourcing system for some time, he can simply set *σ*_*w*_(*t*) to 0, making ${{\rm{\Psi }}}_{w}(t)\leqslant 0$ until he changes his mind.

**Algorithm 2:**
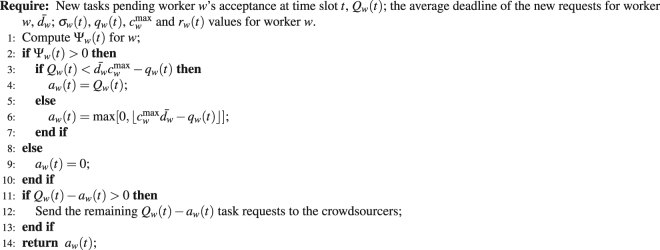
The Distributed Implementation of SMVM.

## Results

In this section, we analyze the performance of SMVM from various perspectives. Firstly, we establish the theoretical performance bounds of SMVM through analysis using standard queueing theory techniques. Secondly, we perform high resolution simulations with worker agent behaviours derived from a large-scale real-world dataset to compare SMVM against four existing approaches. Finally, we present the results from our empirical study comparing SMVM against various human strategies under different conditions.

### Analytical Results

In this section, we analyse the performance bounds of SMVM when the overall workload does not exceed the workers’ maximum collective productivity. Let *U* ^*^ be the utility achieved by an oracle strategy which can perfectly predict the workers’ behaviours in a given crowdsourcing system. There exist constants *σ*, *ε* and *ζ* at a given time slot *t* such that:

11 $$\sigma U(t)-{\rm{\Delta }}({\bf{q}}(t))\geqslant \sigma {U}^{\ast }+\varepsilon \,\sum _{w=1}^{N}{q}_{w}(t)-\zeta .$$

Based on equations (2) and (5), we have:

12 $$\sigma \,\sum _{w=1}^{N}{\mathbb{E}}\{{r}_{w}(t){a}_{w}(t)\}-{\mathbb{E}}\{L({q}_{w}(t+1))-L({q}_{w}(t))\}\geqslant \sigma {U}^{\ast }+\varepsilon \,\sum _{w=1}^{N}{\mathbb{E}}\{{q}_{w}(t)\}-\zeta .$$

Summing both sides of equation (12) over all *t* ∈ {0, 1, …, *T* − 1} yields:

13 $$\sigma \,\sum _{t=1}^{T-1}\sum _{w=1}^{N}{\mathbb{E}}\{{r}_{w}(t)\,{a}_{w}(t)\}-{\mathbb{E}}\{L({q}_{w}(T))-L({q}_{w}(0))\}\geqslant \sigma T{U}^{\ast }+\varepsilon \,\sum _{t=1}^{T-1}\sum _{w=1}^{N}{\mathbb{E}}\{{q}_{w}(t)\}-T\zeta .$$

Since ${q}_{w}(t)\geqslant 0$ for all *w* and *t*, $L(\cdot )\geqslant 0$ and *L*(0) = 0, equation (13) can be re-written as:

14 $$\frac{1}{T}\,\sum _{t=1}^{T-1}\sum _{w=1}^{N}{\mathbb{E}}\{{r}_{w}(t)\,{a}_{w}(t)\}\geqslant {U}^{\ast }-\tfrac{\zeta }{\sigma }+\tfrac{\varepsilon }{\sigma T}\,\sum _{t=1}^{T-1}\sum _{w=1}^{N}{\mathbb{E}}\{{q}_{w}(t)\}+\tfrac{1}{\sigma T}{\mathbb{E}}\{L({q}_{w}(T))\}\geqslant {U}^{\ast }-\tfrac{\zeta }{\sigma }.$$

Therefore, we prove that the *lower bound* on the time-averaged expected utility from workers following SMVM is within $O(\tfrac{1}{\sigma })$ of that achieved by the theoretical optimal solution. As *σ* increases, more task requests will be handled by workers who have demonstrated good reliability in the past, thereby resulting in a higher expected quality of results.

However, the increase in the expected quality of results obtained by increasing *σ* comes at a price. Since ${U}^{\ast }\geqslant 0$, re-arranging the terms in equation (14) yields:

15 $$\begin{array}{lll}\frac{1}{T}\,\sum _{t=1}^{T-1}\sum _{w=1}^{N}\,{\mathbb{E}}\{{q}_{w}(t)\} & \leqslant  & \frac{\sigma }{\varepsilon T}\,\sum _{t=1}^{T-1}\sum _{w=1}^{N}\,{\mathbb{E}}\{{r}_{w}(t)\,{a}_{w}(t)\}-\frac{\sigma }{\varepsilon }{U}^{\ast }+\frac{\zeta }{\varepsilon }-\frac{1}{\varepsilon T}\,{\mathbb{E}}\{L({q}_{w}(T))\}\\  & \leqslant  & \frac{\sigma }{\varepsilon T}\,\sum _{t=1}^{T-1}\sum _{w=1}^{N}\,{\mathbb{E}}\{{r}_{w}(t){a}_{w}(t)\}+\frac{\zeta }{\varepsilon }.\end{array}$$

Therefore, we prove that by following SMVM, the *upper bound* on the time-averaged pending task queue lengths for workers is directly proportional to *σ*. Overall, a larger *σ* value will result in improved quality of results for the tasks, but longer delays in obtaining these results due to a smaller group of highly reliable workers being more heavily loaded.

Due to workers’ physical limitations in a practical crowdsourcing system, when the increase in *σ* starts to cause the expected completion time of tasks to exceed the stipulated deadlines, the total expected utility will drop as the perceived performance of the group of highly reliable workers deteriorates, resulting in them being excluded from future task allocation attempts. Therefore, setting the value of *σ* arbitrarily high will not make the performance of SMVM approach *U* ^*^ indefinitely. The trade-off between task result quality and timeliness only exists within a limited range of the *σ* value. The actual range depends on the workers’ physical limitations in a given crowdsourcing system.

### Computational Results

To evaluate the performance of SMVM under realistic settings, it is compared against four existing approaches through extensive simulations under a crowdsourcing system based on the *Epinions* dataset^40^. This real-world dataset allows us to construct realistic scenarios to perform the comparisons. The simulations illustrate the behaviour of SMVM under different situations. The dataset used in our simulations, together with a readme file explaining how to interpret its contents, can be found at http://dx.doi.org/10.7303/syn7821443.

**Figure 1:**
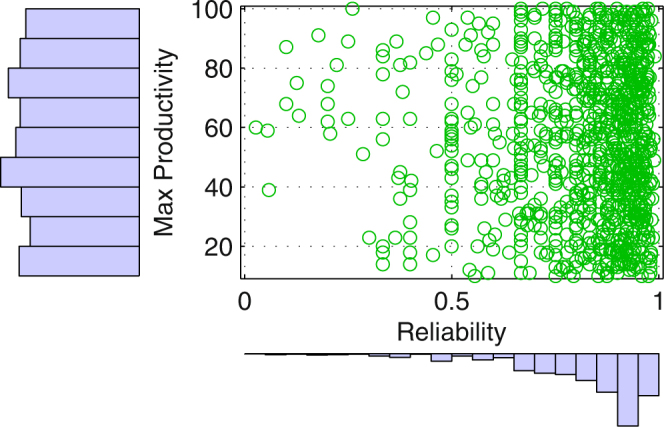
Experiment settings: the distribution of the reliability and the maximum productivity values of the 1,000 agents.

In order to study the performance of SMVM, we generate 1,000 worker agents (WAs). The reliability value for each WA is extracted from the Epinions dataset. It controls the probability for a WA to produce a correct result for a task. The maximum productivity values are randomly generated following a uniform distribution between 10 and 100 tasks per time slot. The distributions of the reliability values and the maximum productivity values of the WAs are illustrated in Fig. 1.

We adopt the load factor (LF) metric from our previous work^25^ to represent the overall workload placed on a crowdsourcing system. It is computed as the ratio between the number of tasks allocated among the worker agents during time slot *t*, *W*_*req*_(*t*), and the maximum collective productivity of the system, Ω $({\rm{i}}{\rm{.}}{\rm{e}}{\rm{.}}\,LF=\tfrac{{W}_{req}(t)}{{\rm{\Omega }}})$. During the experiment, the load factor is varied from 5% to 100% in increments of 5%. In order to control the behaviour settings for all WAs with ease, we set all *σ*_*w*_(*t*) to *σ*. The *σ* value is varied from 5 to 100 in increments of 5 to simulate different quality-timeliness trade-off preferences. The reputation threshold *r*_min_ is set to 0.6. Under each LF setting, the simulation is run for *T* = 10,000 time steps. In all experiments, WAs process tasks at an average rate of $0.9{c}_{w}^{{\rm{\max }}}$ with a standard deviation of $0.1{c}_{w}^{{\rm{\max }}}$. A task must be completed within 1 time slot after it is first assigned to a WA. Thus, the *n* value is set to 1.

In the experiments, we assume that the outcome for each task is binary (i.e., a task is regarded as *successful* if the worker agent produces the correct result before the stipulated deadline; otherwise, it is considered *unsuccessful*). Five duplicate WA populations are created to study the relative performance of the five approaches:

1. The Reputation-based approach (Rep): the probability for a WA to be selected is determined by its reputation standing among all candidate WAs following the softmax choice rule^41^.
2. The Load-balancing approach (LB): tasks are uniformly distributed among the candidate WAs regardless of their reputation.
3. The hybrid approach (Rep + LB): the probability for tasks to be delegated to a WA is adjusted following the approach proposed in Grubshtein *et al*.^19^ which combines reputation-based considerations with load-balancing.
4. The Primal Approximation Algorithm (PAA): workers are matched to tasks based on their skill levels and the values of the tasks while ensuring each worker’s maximum capacity is not exceeded^21^.
5. The SMVM approach: tasks are distributed among candidate WAs following the proposed approach.

The WAs’ reputations are tracked separately in the five agent populations using the BRSEXT method^34^. The relative performance of the five approaches are compared using two main metrics:

1. The average task failure rate which is computed as the percentage of tasks completed before the deadlines by a given approach but with incorrect results; and
2. The average task expiry rate which is computed as the percentage of unsuccessful tasks due to failure to meet task deadlines among all tasks assigned to workers by a given approach.

The average task success rate is thus (100% - the average task failure rate - the average task expiry rate).

**Figure 2:**
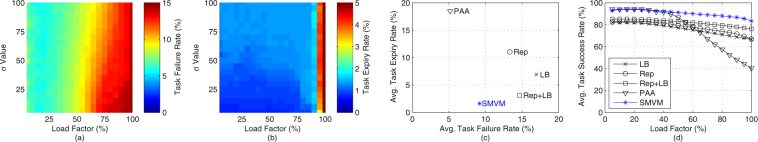
Simulation results: (**a**) task failure rates achieved by SMVM averaged over all simulation time steps under different LF and *σ* settings; (**b**) task expiry rates achieved by SMVM averaged over all simulation time steps under different LF and *σ* settings; (**c**) the task expiry rates vs. the task failure rates averaged over all LF settings and over all simulation time steps for all five approaches; (**d**) the task success rate averaged over all time steps for all five approaches under different LF settings.

Figure 2 shows the experiment results evaluating SMVM. Figure 2(a) shows the task failure rate (TFR) under SMVM. It can be observed that as *σ* increases (i.e. more emphasis is placed on obtaining high quality results from reputable WAs), the TFR decreases as predicted by our theoretical analysis. In addition, as LF increases, WAs with high reputation cannot process the increase in overall workload and an increasing number of less reputable WAs needs to be mobilized. Thus, the TFR increases with increasing LF. Figure 2(b) shows the task expiry rate (TER) under SMVM, or in other words the percentage of tasks not completed before their deadlines. Overall, SMVM is able to maintain very low TERs under most LF and *σ* value settings. The TER increases when LF approaches 100%. As *σ* increases, TER increases as predicted by our theoretical analysis.

Figure 2(c) shows the TFR and TER values averaged over all LF and *σ* value settings for the five approaches. SMVM achieves the lowest TER and the second lowest TFR among all five approaches. Instead of trading a higher TFR for a lower TER like Rep + LB, SMVM is able to reduce TFR and TER simultaneously. This result implies that SMVM is advantageous in terms of meeting real-time demands with high quality results^42^. Figure 2(d) shows the average task success rate of all five approaches under different LF conditions. Although PAA achieves the same performance as SMVM under low LF conditions, its performance deteriorates more significantly than other approaches as LF increases. SMVM outperforms all other approaches, achieving an average of 10.2%, 16.1%, 18.4% and 19.6% higher success rate compared to Rep + LB, Rep, PAA and LB, respectively. Statistical hypothesis testing using the Student’s *t*-test yields *p*-values less than 0.01 when comparing the average task success rate achieved by SMVM against each of the four comparison approaches. This indicates that the improvements in task success rates achieved by SMVM over the four existing approaches are statistically significant at the 99% confidence level. The advantage of SMVM against Rep and LB is more pronounced under high LF conditions. The task success rate achieved by SMVM averaged over all LF and *σ* settings is 90.4%.

### Empirical Results

In order to study how the SMVM approach performs compared to human strategies, we launched the *Agile Manager* (AM) game platform (http://agilemanager.algorithmic-crowdsourcing.com/). The game puts players into various scenarios of managing a team of virtual WAs with diverse characteristics with the aim to maximize the overall task success rate by making quality-timeliness trade-offs.

The game places players into various situations to experience the challenges facing a crowdsourcer who needs to efficiently delegate tasks to workers with diverse reliability and productivity. The virtual team consists of 10 WAs. The game includes 6 distinct levels, each comprising several iterations. In each iteration, the player must delegate a number of tasks to the WAs to maximize the task success rate. A task is fully defined by its value, difficulty, required effort, and deadline.

A player faces information uncertainty about the WAs’ reliability and productivity just like in practical crowdsourcing situations. Each WA exhibits different characteristics in terms of reliability and maximum productivity. The result of a task produced by a WA can be either *satisfactory* or *unsatisfactory*. The reliability variable affects the probability of a WA producing a satisfactory result for a task assigned to it. The maximum productivity variable affects the number of tasks a WA can complete during each iteration. The status of each WA in one game session (i.e., one game level) is tracked by the game platform using two variables: 1) the WA’s reputation; and 2) the WA’s current workload. Reputation acts as a proxy for reliability and is computed following the BRSEXT approach documented in Yu *et al*.^34^. It can be interpreted as the probability of a task being successfully completed by the WA before its deadline. It is displayed in the form of the emotional expression of a WA and a five star scale. The current workload is displayed as the percentage of the maximum productivity of a WA. If a WA is overloaded, the current workload bar never exceeds the 100% marker.

Each of the six game levels implements a distinct setting, as illustrated in Table 1. The *Quality*-*Quantity* (*QQ*) *Trade*-*off* variable is used to control the characteristics of the WAs. If its value is set to “+1”, WAs’ reliability and maximum productivity values are positively correlated. If its value is set to “−1”, WAs’ reliability and maximum productivity values are negatively correlated. Through controlling the number of tasks which need to be assigned to WAs, the *overall workload* relative to the WA team’s productive capacity has been divided into *Medium* (Levels 1 and 2), *High* (Levels 3 and 4) and *Overload* (Levels 5 and 6) cases. At the end of each game session, a player is required to report the strategy he used during the game session. He can choose a mixture of available descriptive options and/or provide his own textual description. The SMVM approach was incorporated into the AM game. It controls another team of WAs with exactly the same characteristics to compete against human players. It faces the same information uncertainty as the players do.

**Table 1:** Game Level Settings.

QQ Trade-off	+1	−1
LF		
Medium	Game Level 1	Game Level 2
High	Game Level 3	Game Level 4
Overload	Game Level 5	Game Level 6

A total of 1,144 participants took part in the study and agreed to share their data with the research community. Among them, 356 are female and 788 are male. In terms of geographic locations, 845 participants are in Singapore while 299 are in China. The participants completed 9,043 valid game sessions. Comprehensive methodological details about the study and links to the dataset can be found in Yu, H. *et al*.^43^. Preliminary analysis of the dataset can be found in Yu, H. *et al*.^44^.

**Figure 3:**
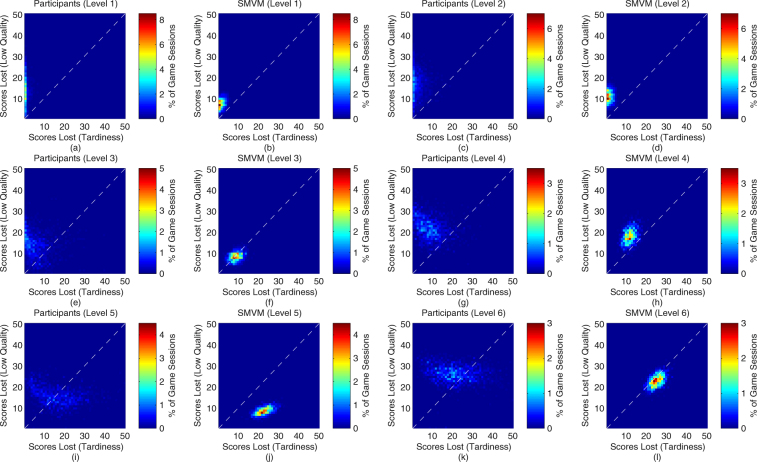
Distributions of scores lost by human players and SMVM (scores are in the range of 0 to 100): (**a**,**c**,**e**,**g**,**i**,**k**) illustrate the distributions of the scores lost by the participants due to low quality of work and failure to meet deadlines in game levels 1–6, respectively. (**b**,**d**,**f**,**h**,**j**,**l**) Illustrate the distributions of the scores lost by SMVM due to low quality of work and failure to meet deadlines in game levels 1–6, respectively. The higher the game level, the more challenging it is for decision-making.

Figure 3 shows the distributions of the scores lost by the human players and SMVM under various conditions due to low quality of work (y-axis) or tardiness (i.e. failure to meet task deadlines) (x-axis). The white dotted diagonal line divides each figure into two regions: 1) above the line: more scores are lost due to low quality work than tardiness, and 2) below the line: more scores are lost due to tardiness than low quality work. In general, it can be observed that the score losses of SMVM are within a clearly defined tight range, whereas the score losses by human players span wider ranges due to the diverse strategies adopted and possible individual differences in executing those strategies. Under game levels 1, 3, and 5 in which the WAs’ reliability values are positively correlated to their maximum productivity values, SMVM trades higher scores lost due to tardiness for lower scores lost due to low quality work to deal with the increase in workload. Under game levels 2, 4, and 6, the WAs’ reliability values are negatively correlated to their maximum productivity values. This case is more challenging as WAs which produce higher quality work can process a smaller number of tasks per iteration. Under these conditions, SMVM sacrifices both scores lost due to low quality work and tardiness to deal with the increase in workload. A similar trend can be observed in human performance but to a more pronounced extent compared to SMVM.

**Figure 4:**
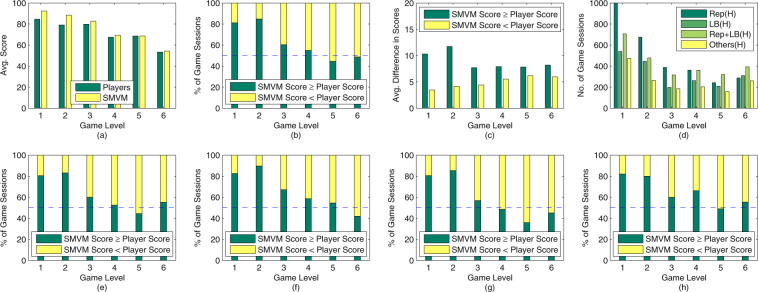
Summary of results comparing SMVM with human strategies: (**a**) the average scores achieved by SMVM and the players across the 6 game levels; (**b**) the relative performance between SMVM and players across the 6 game levels; (**c**) the average difference in scores in game sessions in which SMVM outperforms/underperforms players across the 6 game levels; (**d**) the number of game sessions in which players adopt each of the major categories of strategies: *Rep*(*H*), *LB*(*H*), *Rep* + *LB*(*H*) and *Others*(*H*); (**e**) the relative performance between SMVM and players adopting Rep(H); (**f**) the relative performance between SMVM and players adopting LB(H); (**g**) the relative performance between SMVM and players adopting Rep + LB(H); (**h**) the relative performance between SMVM and players adopting Others(H).

Figure 4 summarizes the results of comparing SMVM against various human strategies across the six game levels. Figure 4(a) shows the average scores achieved by SMVM and human players in each game level. Under medium and high workload conditions, SMVM outperforms human players, although the advantage of SMVM is less pronounced when the QQ trade-off variable is set to −1. Under overload conditions, human performance is on par with that of SMVM in terms of average scores. In terms of the percentage of game sessions in which SMVM beat human scores (Fig. 4(b)), SMVM performed significantly better than humans under medium and high workload conditions, but worse than humans under overload conditions. Despite this, the average scores achieved by SMVM is never less than humans’ (Fig. 4(a)) because when SMVM achieves higher scores than the human players, the average difference in the scores is significantly higher than cases in which SMVM achieves lower scores than human players (as shown in Fig. 4(c)).

In the game, there are five strategy choices available for self-reporting. In total, there are 2^5^ − 1 = 31 combinations of these strategy choices. Among them, the most widely adopted strategies are: assigning more tasks to reputable Was − *Rep*(*H*) (adopted 33% of the time), balancing the workload among the WAs − *LB*(*H*) (adopted 22% of the time), and combining Rep(H) with LB(H) − *Rep* + *LB*(*H*) (adopted 28% of the time). The remaining 28 strategy combinations, which we label as *Others*(*H*), were employed 17% of the time. The label “(H)” is added to indicate that these strategies are executed by humans.

Figure 4(d) shows the distribution of the four main human strategies across the six game levels. It can be observed that Rep(H) and Rep + LB(H) are the two most often adopted strategies under all conditions. As workload increases, Rep + LB(H) gradually becomes the most dominant human strategy. Others(H) is the least adopted among these four strategies across all levels. There is a significant number of game sessions in which players adopted Rep + LB(H). Below are sample quotes from players’ descriptions which can shed some light on how they executed the Rep + LB(H) strategy:

- “*I assigned more tasks to workers with more stars*, *but when they didn*’*t finish the tasks in last sprint*, *I assigned fewer tasks to them*, *and I will assign fewer tasks to workers with fewer stars and workers with more workload*.”
- “*High difficulty and high value tasks are given to workers with more stars*; *easy or short jobs are given to workers with fewer stars*.”
- “*Day 1*, *assign equally to everyone*. *Day 2*, *identify those that are incompetent*. *Day 3*, *identify those that cannot take too many tasks and give them fewer*. *Day 4*, *if there is any one that cannot take too much*, *assign to those that are more capable*. *Day 5*, *same as Day 4*.”

From Fig. 4(e), SMVM significantly outperforms Rep(H) under medium and high workload conditions. Under overload conditions, when WAs’ reliability values are positively correlated to their maximum productivity values (in Level 5), Rep(H) performs better than SMVM 56% of the time. This is because under such a condition, assigning more tasks to WAs with high reputation results in both high quality and timely completion. However, when these two characteristics are negatively correlated (in Level 6), SMVM outperforms Rep(H) 55% of the time. On average, SMVM outperforms Rep(H) 70% of the time. A similar trend can be observed in Fig. 4(f). However, instead of outperforming LB(H) in Level 6, SMVM outperforms LB(H) 55% of the time in Level 5. On average, SMVM outperforms LB(H) 70% of the time. In Level 6, WAs with high reputation work slower and balancing workload among more WAs is advantageous. As shown in Fig. 4(g), SMVM outperforms Rep + LB(H) under medium workload and high workload without QQ trade-off. Starting from high workload with QQ trade-off conditions (i.e. Level 4), human players adopting Rep + LB(H) outperform SMVM. On average, SMVM outperforms Rep + LB(H) 63% of the time. As shown in Fig. 4(h), SMVM outperforms players adopting Others(H) 69% of the time. Over the 9,043 game sessions, SMVM outperforms human players 68% of the time. Nevertheless, under Medium workload conditions, SMVM outperforms players 83% of the time. As the overall workload in practical crowdsourcing systems (such as mTurk) is often in the medium range^13^, SMVM can significantly improve the efficiency of such existing crowdsourcing systems.

## Discussion

In this paper, we proposed, analyzed, and evaluated a task delegation approach – SMVM – which influences individual task allocation decisions in order to achieve desirable overall system characteristics in crowdsourcing. The proposed approach yields new insights into the emergent collective behaviour in crowdsourcing in the form of the WDI. With the WDI, the proposed SMVM approach optimizes system throughput, expected result quality and waiting time based on information about individual workers’ reputation, workload and motivation to work. With this simple index, SMVM optimizes the distribution of tasks among workers to steer a crowdsourcing system towards high collective productivity. It is shown to achieve collective utility which is asymptotically bounded by theoretical optimal solutions if workers adhere to recommendations made by SMVM. SMVM efficiently optimizes the social welfare of crowdsourcing systems with polynomial time complexity. Extensive simulation-based and empirical experiments demonstrate that SMVM is able to simultaneously reduce task expiry rates and task failure rates, thereby enabling algorithmic management to fulfil the real-time demand for crowd productivity with high quality results.

This work opens up a series of interesting research directions. In order to further evaluate SMVM on ecologically valid data, we are incorporating SMVM into a mobile crowdsourcing platform – Silver Productivity^45^ – which helps community groups organize productive aging activities for senior citizens. Here we show that workers’ reputation, workload and motivation to work are crucial factors influencing the emergent collective behaviour of a crowd. The finding can be used to advise machine learning research to focus on designing more accurate data-driven approaches to estimate the ground truth values of these factors in order to help crowdsourcing systems make more efficient use of the human resources. Tracking of workers’ motivation to work can be done through direct self-reports by workers, or indirectly by using statistical analysis methods (such as Kalman Filtering^46^) to track a worker’s past productivity output over a period of time and predict its likely value in the immediate future.

In future research, we will study the impact of workers not adhering to SMVM recommendations on system performance. In addition, as the empirical dataset contains decision data for cases in which human strategies outperform SMVM, it can be a valuable source for exploring advanced computational models which can be even more effective than the proposed approach. In social mobilization, it is more advantageous to influence people’s behaviour through persuasion or incentives rather than simply telling them what to do. By establishing a pattern of behaviour which is desirable for improving overall system performance in the long run, this paper provides the human behaviour research community with a clearly defined objective towards which to steer crowdsourcing workers with incentives^47^ or persuasion techniques^48,49^. We will also investigate how to incorporate the workers’ other human aspects (e.g., mood^50^, collaboration^51^) into the optimization process to improve their user experience.

In conclusion, the proposed approach and results provide a stepping stone towards more efficient management of large-scale crowdsourcing systems based on evidence about workers’ behaviours. They provide insights and represent an important step towards designing stable and scalable resource allocation decision support mechanisms for social and economic systems.
